# Single-cell RNA sequencing reveals Immune Education promotes T cell survival in mice subjected to the cecal ligation and puncture sepsis model

**DOI:** 10.3389/fimmu.2024.1366955

**Published:** 2024-03-18

**Authors:** Steven D. Ham, Mabel N. Abraham, Clifford S. Deutschman, Matthew D. Taylor

**Affiliations:** ^1^ The Division of Critical Care Medicine, Department of Pediatrics, Cohen Children’s Medical Center/Northwell Health, New Hyde Park, NY, United States; ^2^ Sepsis Research Laboratory, The Feinstein Institutes for Medical Research, Manhasset, NY, United States

**Keywords:** cecal ligation and puncture, mouse sepsis model, sepsis, T cell, CD4, CD8, T cell activation, T cell memory

## Abstract

**Background:**

Individual T cell responses vary significantly based on the microenvironment present at the time of immune response and on prior induced T cell memory. While the cecal ligation and puncture (CLP) model is the most commonly used murine sepsis model, the contribution of diverse T cell responses has not been explored. We defined T cell subset responses to CLP using single-cell RNA sequencing and examined the effects of prior induced T cell memory (Immune Education) on these responses. We hypothesized that Immune Education prior to CLP would alter T cell responses at the single cell level at a single, early post-CLP time point.

**Methods:**

Splenic T cells were isolated from C57BL/6 mice. Four cohorts were studied: Control, Immune-Educated, CLP, and Immune-Educated CLP. At age 8 weeks, Immune-Educated and Immune-Educated CLP mice received anti-CD3ϵ antibody; Control and CLP mice were administered an isotype control. CLP (two punctures with a 22-gauge needle) was performed at 12-13 weeks of life. Mice were sacrificed at baseline or 24-hours post-CLP. Unsupervised clustering of the transcriptome library identified six distinct T cell subsets: quiescent naïve CD4^+^, primed naïve CD4^+^, memory CD4^+^, naïve CD8^+^, activated CD8^+^, and CD8^+^ cytotoxic T cell subsets. T cell subset specific gene set enrichment analysis and Hurdle analysis for differentially expressed genes (DEGs) were performed.

**Results:**

T cell responses to CLP were not uniform – subsets of activated and suppressed T cells were identified. Immune Education augmented specific T cell subsets and led to genomic signatures favoring T cell survival in unoperated and CLP mice. Additionally, the combination of Immune Education and CLP effected the expression of genes related to T cell activity in ways that differed from CLP alone. Validating our finding that IL7R pathway markers were upregulated in Immune-Educated CLP mice, we found that Immune Education increased T cell surface IL7R expression in post-CLP mice.

**Conclusion:**

Immune Education enhanced the expression of genes associated with T cell survival in unoperated and CLP mice. Induction of memory T cell compartments via Immune Education combined with CLP may increase the model’s concordance to human sepsis.

## 1 Introduction

Sepsis is defined as life-threatening organ dysfunction caused by a dysregulated host response to infection (1). The disorder affects nearly 50 million people annually, and it is a leading cause of in-hospital mortality and critical illness globally (2, 3). Moreover, it has been suggested that many sepsis-associated deaths are not preventable with current hospital-based care measures (4). This concern underscores the need for innovative sepsis research and novel therapeutic approaches.

Because of its biologic complexity sepsis is best studied *in vivo*, often using animal models. The cecal ligation and puncture (CLP) model is the most commonly used animal model of sepsis because it closely mimics the hemodynamic and metabolic phases of human sepsis (5). However, recent studies comparing laboratory mice kept in specific pathogen-free facilities to mice from “natural” environments have led to findings that may significantly affect the CLP model. These studies demonstrated that laboratory mice with limited antigenic exposure lack diverse memory T cell compartments (6, 7). We have previously shown that administration of exogenous anti-CD3ϵ antibody, a process herein termed Immune Education, to induce T cell memory before subjecting mice to CLP enhanced innate and adaptive immune responses, organ dysfunction, and mortality (8–10).

CD3, which consists of four chains (CD3γ, CD3δ, and 2 CD3ϵ chains), is a T cell coreceptor that complexes with the T cell receptor (TCR) during early T cell activation (11). Prior *in vitro* studies have shown that the Armenian hamster monoclonal antibody clone 145-2C11 targeting CD3ϵ can be used to prime T cells and induce memory T cell responses that are indistinguishable from peptide-primed memory T cells (12). Our prior work demonstrated that Immune Education can be used to induce polyclonal CD4 and CD8 T cell memory populations to partly address the lack of a cadre of memory T cells in CLP (8, 9). In brief, the proportion of CD4 and CD8 memory T cells in spleen, lung, and liver were increased 35 days following a single intravenous administration of a moderate dose of anti- CD3ϵ treatment. T cell memory phenotypes in these mice persisted for up to almost six months. These findings suggest that Immune Education induces heterogenous memory T cell development (9). T cell memory induction through Immune Education thus increased the fidelity of CLP as a model of human sepsis while providing a tool for studying the impact of T cell memory on CLP and, conversely, the effects of CLP on T cell memory responses. Using induced T cell memory via Immune Education allows for examination of the effect of CLP on specific T cell subsets that were previously lacking in laboratory mice. Moreover, induced T cell memory may permit the study of T cell responses to specific cytokine receptor signaling pathways after CLP. The IL7 receptor (IL7R) pathway is of particular interest given its roles in naïve T cell survival and effector to memory T cell transition (13). Because of these functions, there have been trials investigating the therapeutic potential of IL7 in human sepsis (14).

Our study utilized single-cell RNA sequencing (scRNA-seq), which has been crucial to elucidating the heterogeneity of the full T cell repertoire (15). T cells have the ability to differentiate from naïve T cells into memory T cells upon initial antigen exposure and following differentiation gain different functional properties that can drive immune responses. Further, T cells can “remember” the cytokine response during prior infection and will produce a more tailored response on repeat exposure. Many different T cell responses have been described in the literature: CD4 T cells can produce Th1, Th2, Th17, Th22, Th23, T follicular helper, and T_reg_ responses while CD8 T cells have been shown to respond with a Tc1 or Tc2 phenotype (16). There are likely other T cell responses that have not yet been defined as well. Given the immense complexity and plasticity of individual T cell responses during an immune response, scRNA-seq has helped elucidate gene expression patterns across T cell subsets and disease states unable to be captured by “bulk” sequencing approaches (17). We used scRNA-seq to examine T cell subset specific responses to CLP, and specifically interrogated the effects of prior induced T cell memory via Immune Education on these responses.

## 2 Materials and methods

### 2.1 Mice

**Figure 1:**
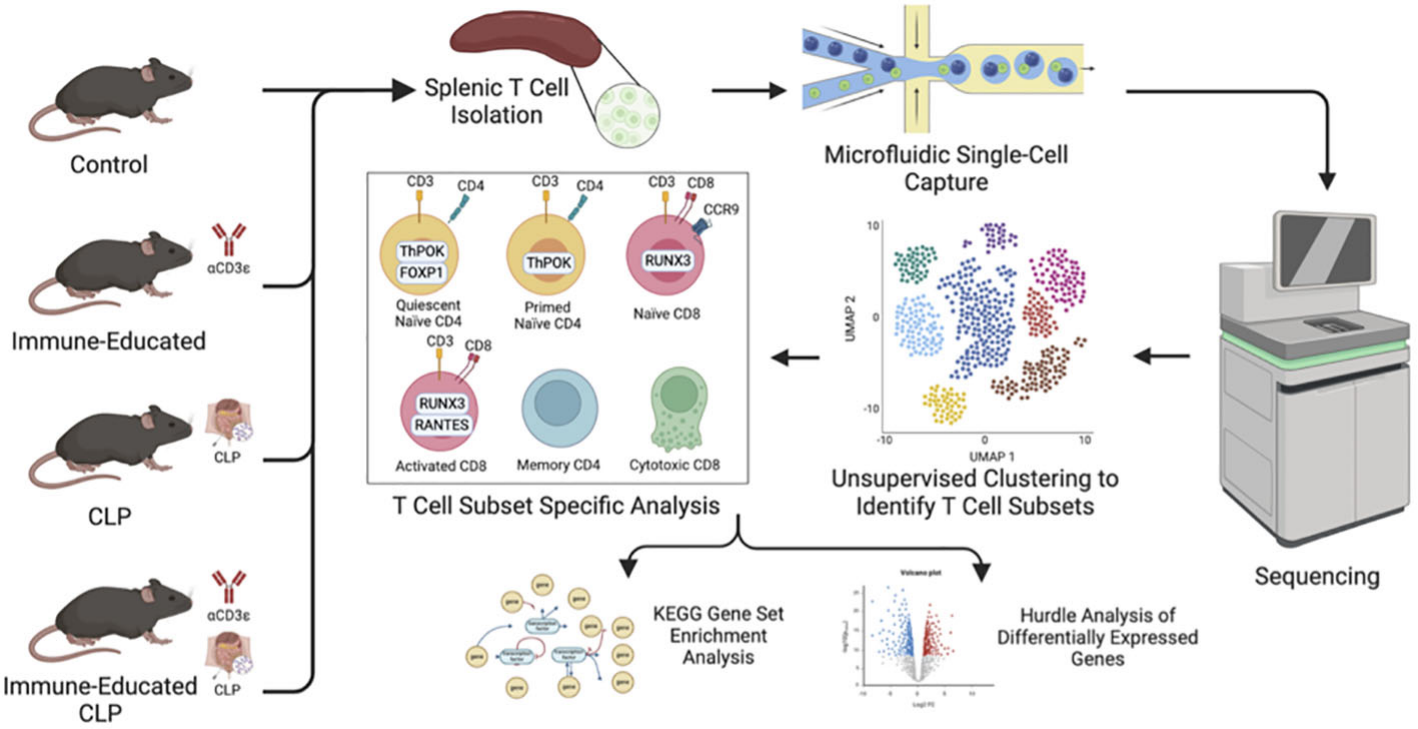
Workflow illustrating mouse cohorts, splenic T cell isolation by negative selection and cell sorting, and single-cell capture via 10x Genomics Chromium Next GEM (gel beds-in-emulsion) prior to sequencing. After unsupervised clustering to identify distinct T cell subsets, cell subset specific analysis was performed with KEGG gene set enrichment analysis and Hurdle analysis to identify differentially expressed genes.

All studies were approved by the Institutional Animal Care and Use Committee (IACUC #2017-039) and adhered to National Institutes of Health and Animal Research: Reporting of *In Vivo* Experiments (ARRIVE) guidelines. C57BL/6J mice were obtained from The Jackson Laboratory (Bay Harbor, ME) and maintained at the animal facility of the Feinstein Institute for Medical Research. We used only male mice in this study for three reasons: 1) previous studies indicated that mortality from CLP is higher in male mice; 2) because the differences in mortality reflect, in part, a protective effect from estrogen, responses in female mice are subject to variability secondary to the estrous cycle; and 3) most of the vast literature on CLP has been generated using male mice only, making comparisons with female mice problematic (18, 19). The mice used for scRNA-seq were split into four cohorts: Control, Immune-Educated, CLP, and Immune-Educated CLP (**Figure 1**).

### 2.2 *In vivo* immune education

Immune Education was performed as previously described (8). Briefly, Ultra-LEAF anti-mouse CD3ϵ antibody (145-2C11, BioLegend, San Diego, CA; 50 μg in 200 μL sterile phosphate-buffered saline) was administered via retro-orbital venous sinus injection to 8-week-old mice (9). Thirty-five days later, this approach had increased the total number and fraction of splenic memory (CD44^+^/CD11a^+^) T cells in both CD4 and CD8 subpopulations (9).

### 2.3 Cecal ligation and puncture procedure

Mice were subjected to CLP with two 22-gauge punctures under isoflurane anesthesia as previously described (20). All mice that underwent CLP were approximately 12-13 weeks of age. Animals were resuscitated with 50 mL/kg sterile saline immediately after surgery through subcutaneous tissue injection. CLP and Immune-Educated CLP mice were euthanized at a single, early post-CLP time point (24-hours after CLP). Antibiotics were not administered because of studies indicating that they alter the early immune responses to CLP (21, 22). Access to food and water was identical for all animals.

### 2.4 Leukocyte isolation

All mice were euthanized either at baseline (Control, Immune-Educated) or at 24 hours post-CLP (CLP, Immune-Educated CLP), at which time spleens were harvested. Single cell suspensions were prepared, and the T cells were sorted by negative selection using a validated pan-T cell isolation kit (Miltenyi Biotec). This technique selects a population of cells that are approximately 90% T cells. The population was increased to approximately 97% purity using fluorescence-assisted cell sorting (BD FACSAria™ III Cell Sorter, San Jose, CA) for live, singlet, CD90^+^ cells to ensure that primarily CD4 and CD8 T cells were analyzed.

### 2.5 10x Genomics single-cell sample processing and RNA sequencing

Libraries were prepared using the 10x Genomics Chromium Next GEM (gel beds-in-emulsion) Single Cell 3’ Reagent Kit per the manufacturer’s instructions. The splenic T cells were loaded on a Chromium Next GEM Chip and then applied to the Chromium Controller, which performed single cell partitioning into the nanoliter-scale GEMs. Each GEM contained an identifying barcode for downstream single cell analysis. These sample libraries were then sequenced using an Illumina Novaseq System following the 10x Genomics next-generation sequencing specifications.

### 2.6 Processing, dimensionality reduction, and clustering of scRNA-seq data

Cell Ranger analysis pipelines were used to demultiplex the raw scRNA-seq data and align it to the reference *Mus musculus* genome (GRCm38). Data files were then uploaded to Partek® Flow®, a cloud-based genomic analysis platform, for analysis. Quality control analysis was performed: cells with <200 detected genes, >5,000 detected genes, and >8% mitochondrial genes were determined to be low quality cells (695 of 9,022 total cells) and filtered out (**Supplementary Figure 1**) (23, 24). Features not expressed in at least 99.9% of cells (5,903 of 20,085 total genes) and ribosomal features (101 genes of 20,085 total genes) were filtered out prior to normalization. Primary component analysis (PCA) was used for initial dimensionality reduction. Graph based clustering with the top 20 primary components (PCs) was performed using a Louvain algorithm and resolution of 1.0. Graph based clusters were then used to define cell populations on Uniform Manifold Approximation and Projection (UMAP) plots. GraphPad Prism 10 was used to perform two-way ANOVA and multiple comparisons testing with Šídák correction to compare cell counts in T cell subsets across the experimental conditions.

### 2.7 Gene set enrichment and differential gene expression analyses

Gene set enrichment analysis (GSEA) analyzes sequencing data by determining if there are statistically significant differences in the expression of predefined gene sets between two biologic phenotypes. GSEA was used to identify altered biochemical pathways as described in the Kyoto Encyclopedia of Genes and Genomes (KEGG) pathway database. This compendium includes manually created pathways derived from published literature to depict molecular interactions (25). Pathways were considered significant at a false discovery rate (FDR) ≤0.25 and P-value ≤0.05. Cytoscape v3.10.1 was used to perform network analysis (26). Additionally, hurdle model analyses were performed on the top 2,000 features with highest variance to identify differentially expressed genes (DEGs) in each T cell subset (27). The hurdle model is widely used for scRNA-seq analysis because it accounts for zero inflation (a distribution where there are frequent zero counts i.e., cells not expressing a certain gene), which is commonly seen with scRNA-seq data (28). Statistical significance for DEGs, genes with differences in read counts between two experimental conditions, was defined by P-value ≤0.01 and fold change of <-2 or >2.

### 2.8 Flow cytometry analysis

Flow cytometry was performed on a BD LSR Fortessa 16-color cell analyzer or a BioRad ZE5 16-color analyzer and analyzed using FlowJo software version 10 (BD Bioscience, San Jose, CA). Staining was performed with the following antibodies: CD90.2, CD8a, CD4, and CD127.

## 3 Results

### 3.1 Identification of six distinct T cell subsets via unsupervised cluster analysis of the single cell RNA sequencing library

Our scRNA-seq library consisted of 9,022 single cells and 20,097 total features from the 12 samples. Following data quality checks our library consisted of 14,081 features from 8,327 high quality cells. Cells that highly expressed pro-B cell markers (82 cells), NK cell markers (431 cells), and lncRNAs (1,188 cells) were filtered out. Additionally, *Cd3e ^–^* cells (668 cells) were also omitted leaving 5,958 *Cd3e^+^* cells for final analysis. Control mice had 959 cells (median counts per cell 12,822), Immune-Educated mice had 2,052 cells (median counts per cell 12,857), CLP mice had 1,154 cells (median counts per cell 11,677), and Immune-Educated CLP mice had 1,793 cells (median counts per cell 11,966).

**Figure 2:**
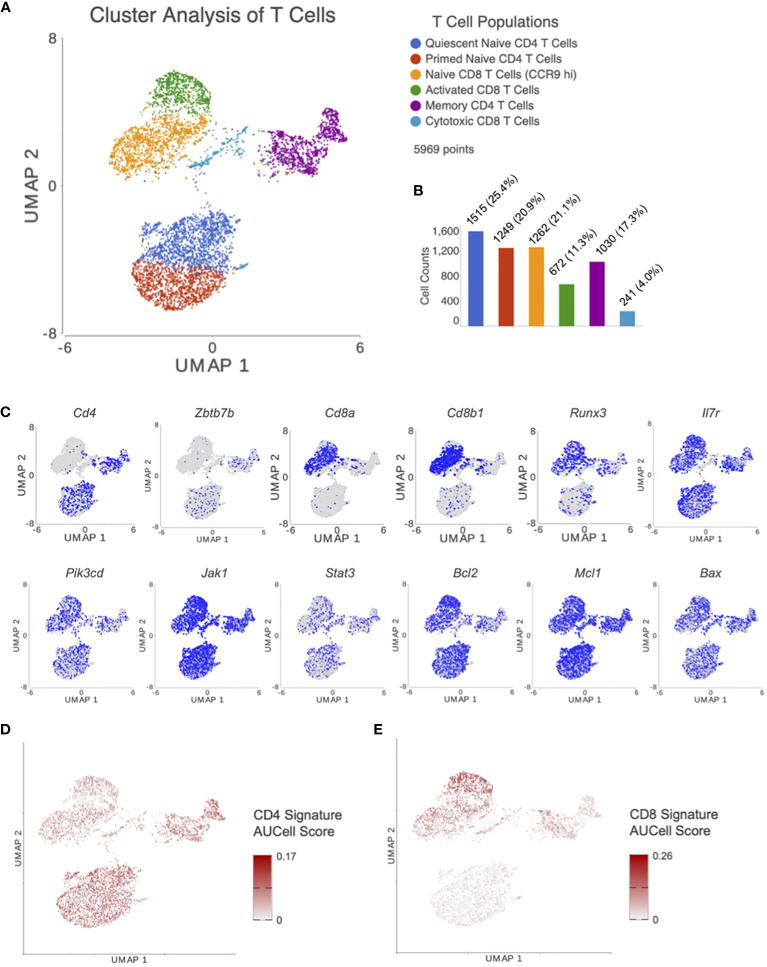
After processing and normalizing the scRNA-seq library, dimensionality reduction was performed using PCA. **(A)** Following unsupervised graph based cluster analysis, UMAP plots were created to identify 6 distinct T cell subsets. **(B)** A bar graph was generated depicting the number of cells in each T cell subset. **(C)** Expression levels for genes of interest were visualized with UMAP. Genes included were markers for CD4 T cells (*Cd4, Zbtb7b*) and CD8 T cells (*Cd8a, Cd8b1, Runx3*), and those involved in the IL7R signaling pathway (*ll7r, Pik3cd, Jak1, Stat3*, and *Bcl2* family members). AUCell was performed using previously published gene signatures for **(D)** CD4 and **(E)** CD8 T cells. These results confirm appropriate clustering of CD4 and CD8 T cells based on previous results external to our dataset.

Cluster analysis on UMAP plot, using T cells from all treatment groups, revealed 6 distinct T cell subsets which were classified by canonical cell marker genes as seen in **Figure 2A**. The top 25 differentially expressed features in each cluster are listed in **Supplementary Table 1**. Clusters 1 and 2 (*Cd3* ^+^, *Cd4* ^+^, *Zbtb7b* ^+^) were identified as a naïve CD4 subpopulation. Cluster 1 was noted to have increased *Foxp1* expression, a marker of CD4 T cell quiescence (29), when compared to cluster 2. Thus, cluster 1 was identified as quiescent naïve CD4 T cells and cluster 2 was identified as primed naïve CD4 T cells. Clusters 3 and 4 (*Cd3* ^+^, *Cd8a* ^+^, *Cd8b* ^+^, *Runx3* ^+^) were identified as CD8 T cells. Cluster 3 was noted to have higher expression of genes characteristic of naïve CD8 T cells such as *Ccr9* (30). On the other hand, cluster 4 was consistent with an activated CD8 T cell population with higher fold levels of expression in genes such as *Ccl5* (*Rantes*) and *Xcl1* (*Atac*) (31). Cluster 5 was a very diverse cluster of memory CD4 T cells (*Cd3* ^+^, *Cd4* ^+^) including T_reg_ (*Foxp3* ^+^), Th1 (*Tbx21* ^+^), Th2 (*Gata3* ^+^), and Th17 (*Rorc* ^+^) cells. Lastly, cluster 6 had *Cd3* ^+^, *Cd4* ^-^, *Cd8a* ^lo^, and *Cd8b* ^lo^ T cells that expressed cytotoxic lymphocyte markers such as *Eomes*, which was consistent with a CD8 cytotoxic T cell population.

The distribution of cells in each subset can be seen in **Figure 2B**. Distribution of genes involved in CD4-CD8 lineage differentiation and IL7 signaling for T cell survival/apoptosis were plotted on UMAP plots for visualization (**Figure 2C**). AUCell, an analysis tool using “Area Under the Curve” (AUC) to calculate the enrichment of active gene sets, was performed to validate the identified T cell subsets from unsupervised clustering in our UMAP plots. AUCell results demonstrated whether CD4 (**Figure 2D**) and CD8 (**Figure 2E**) signatures from Chopp, et al. (32) were within the top 5% of ranked genes from each cell. These results confirm appropriate clustering of CD4 and CD8 T cells based on previous results external to our dataset.

### 3.2 Immune Education augments cell counts in naïve CD4 T cell subsets

**Figure 3:**
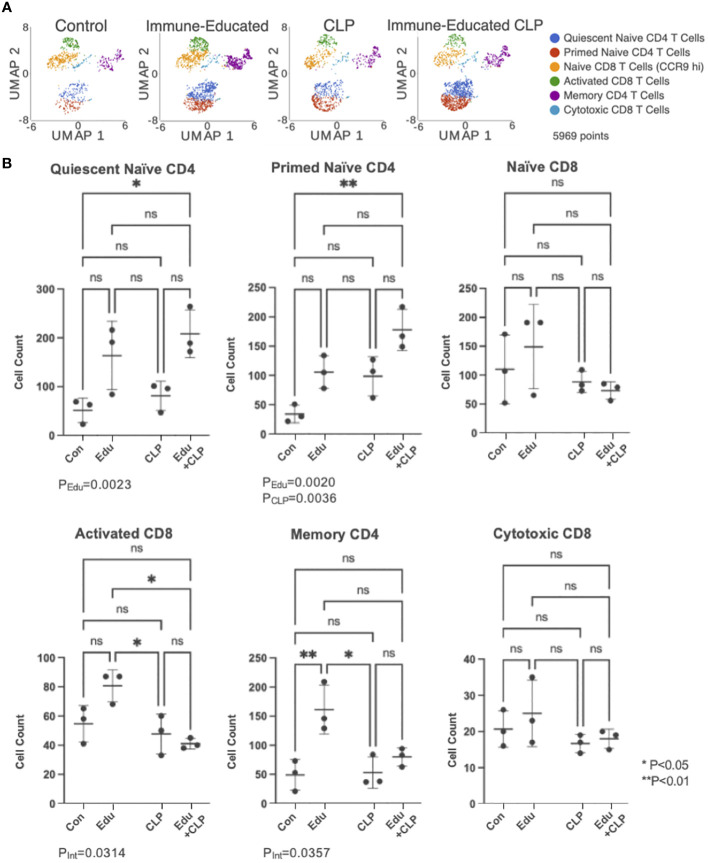
**(A)** UMAP plots of cells in each of the experimental groups were created for visualization. **(B)** Cell counts within each T cell subset were then graphed on dot plots (Con=Control, Edu-Immune-Educated, CLP=CLP, and Edu+CLP=Immune-Educated CLP). Two-way ANOVA and Šídák’s multiple comparisons test were performed to examine differences in T cell subset sizes across the experimental groups. Significance was indicated by asterisks, and significant P-values were listed under the plots. ns, not significant.

The UMAP plot from **Figure 2A**, depicting T cell subsets identified by unsupervised cluster analysis, was split by experimental group in **Figure 3A**. This allowed for visualization of T cell subset sizes across the treatment conditions. The cell counts within each T cell subset across experimental groups were graphed on box plots (**Figure 3B**) with significance indicated based on results from two-way ANOVA and Šídák’s multiple comparisons test.

In the quiescent naïve CD4 T cell subset, Immune Education led to an increase in cell counts (P_Edu_=0.0023). For the cell counts in the primed naïve CD4 T cell subset, both Immune Education (P_Edu_=0.0020) and CLP (P_CLP_=0.0036) led to statistically significant increases, but the interaction term was not statistically significant (P_Int_=0.8245).

In the activated CD8 T cell subset, the interaction term was statistically significant (P_Int_=0.0314) indicating that the effect of CLP differs in control and Immune-Educated mice. Immune-Educated mice had significantly more activated CD8 T cells than Immune-Educated CLP (P=0.0124) mice. The interaction term was also significant on analysis of cell counts in the memory CD4 T cell subset (P_Int_=0.0357). In the memory CD4 T cell subset, Immune-Educated mice had more memory CD4 T cells when compared to Control (P=0.0093); the number of memory CD4 T cells was lower following CLP in Immune Educated mice.

No statistically significant changes were noted in the naïve CD8 and cytotoxic CD8 T cell subpopulations.

### 3.3 Genomic signatures of immune-educated mice favor T cell survival and clonal proliferation when compared to control mice

A list of all significant KEGG pathways—manually created gene pathways depicting biological processes from published literature—in Immune-Educated mice relative to Control mice across the T cell subsets can be found in **Supplementary Table 2**. A list of all significant DEGs—genes that have differential read counts across two experimental conditions— in Immune-Educated mice relative to Control mice across the T cell subsets can be found in **Supplementary Table 3**.

**Figure 4:**
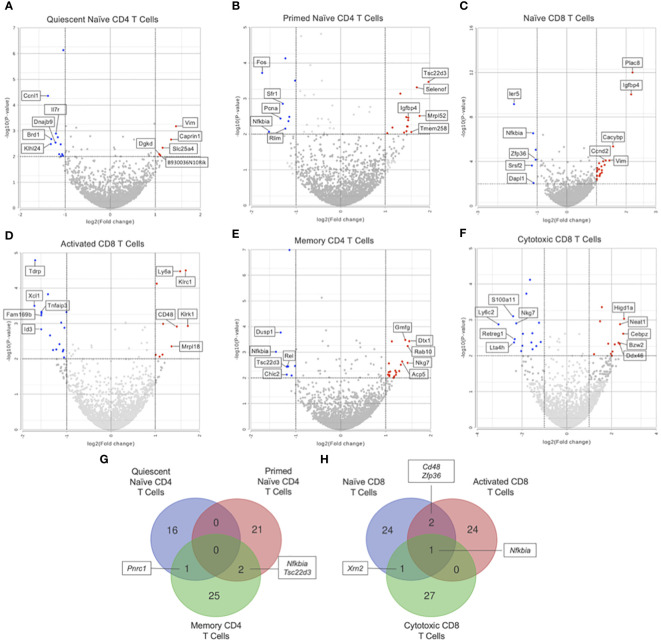
Differential gene expression by hurdle analysis was performed within each T cell subset to compare the effect of Immune-Education relative to Controls. Volcano plots were used to show the adjusted P values and log2 fold change values from these analyses. Red dots indicate upregulated genes, blue dots indicate downregulated genes, and gray dots represent insignificant or inconclusively different genes in Immune-Educated mice relative to Control mice. The top 5 upregulated and top 5 downregulated genes are labeled on the volcano plots. Results for **(A)** quiescent naïve CD4, **(B)** primed naïve CD4, **(C)** naïve CD8, **(D)** activated CD8, **(E)** memory CD4, and **(F)** cytotoxic CD8 T cells are shown. The full list of DEGs can be found in **Supplementary Table 3**. Overlapping DEGs in the **(G)** CD4^+^ subsets and **(H)** CD8^+^ subsets were identified and depicted on Venn diagrams.

GSEA of naïve CD4 T cells (clusters 1 and 2) showed no significant differences in KEGG pathways between Immune-Educated and Control mice. Hurdle analysis of quiescent naïve CD4 T cells (cluster 1) identified 17 DEGs; following Immune Education, expression in 5 was upregulated while expression in 12 was downregulated (**Figure 4A**). Among the genes whose expression were affected by Immune Education were *Il7r* (downregulated 2.31 fold, P=0.0013) and *Bcl2* (B-cell lymphoma-2; upregulated 2.30 fold, P=0.0108), whose encoded protein limits apoptosis and enhances cell survival by promoting IL7 mediated processes (33, 34). This may indicate IL7-mediated cell survival via Bcl2 in the quiescent naïve CD4 T cells in Immune-Educated mice. Hurdle analysis of primed naïve CD4 T cells (cluster 2) identified 23 DEGs; following Immune Education, expression in 14 was upregulated while expression in 9 was downregulated (**Figure 4B**). Among the genes whose expression were affected by Immune Education were *Bcl2* (upregulated 2.48 fold, P-value 0.0349) and *Nfkbia* (downregulated 3.05 fold, P=0.0086), whose protein IκB-α (inhibitor kappa B-alpha) inhibits NF-κB (nuclear factor kappa-light-chain-enhancer of activated B cells). It has been suggested that IκB-α mediated inhibition of NF-κB signaling impairs T cell proliferation (35, 36). These results suggest enhanced cell survival in the primed naïve CD4 T cell clusters following Immune Education. Additionally, the expression of *Igfbp4* (insulin-like growth factor binding protein 4; upregulated 2.87 fold, P=0.0034) was also affected by Immune Education in the primed naïve CD4 T cell subset, which is notable since IGFBP-4 signaling has been associated with preferential CD4 effector Th17 differentiation (37). The importance of Th17 cells in host defense against pathogens, especially at mucosal surfaces, has been well established (38). Lastly, in primed naïve CD4 T cells, Immune Education affected expression of *Cd48* (*Slamf2*; upregulated 2.78 fold, P=0.0033). Notably CD48, in conjunction with its ligand 2B4, has co-stimulatory functions during CD3-induced T cell proliferation, interacts with CD2 during T cell activation, and is upregulated during inflammation (39).

In the naïve CD8 T cell subset (cluster 3), a total of 65 KEGG pathways were upregulated in Immune-Educated mice when compared to Controls (there were no significantly downregulated pathways). Among the enriched gene sets following Immune Education was the JAK-STAT signaling pathway (NES 1.60) which is significant given its central role in cytokine responsiveness. As an example, IL15 has been shown to promote CD8 T cell proliferation by activating the JAK-STAT signaling pathway which upregulates Bcl-2 (40, 41). Hurdle analysis of naïve CD8 T cells identified 27 DEGs; following Immune Education, expression in 21 was upregulated while expression in 6 was downregulated (**Figure 4C**). Among the impacted genes was *Cd5* (upregulated 2.42 fold, P=0.0002), which is notable because peripheral CD5^hi^ naïve CD8 T cells have been shown to undergo more efficient clonal recruitment and expansion in response to foreign antigens relative to CD5^lo^ naïve CD8 T cells (42). Moreover, CD5^hi^ CD8 T cells have been shown to have higher propensity for self-reactivity (43). Additionally, expression of *Cd7* (upregulated 2.42 fold, P=0.0002) differed following Immune Education, which is notable given the costimulatory role of CD7 during TCR signaling (44, 45). Lastly, Immune Education affected expression of *Nfkbia* (downregulated 2.19 fold, P<0.0001), which suggests increased CD8 T cell proliferation following Immune Education (35).

Within the activated CD8 T cell subset (cluster 4), 7 KEGG pathways were upregulated in Immune-Educated mice when compared to Controls. Among the activated KEGG pathways following Immune Education was natural killer cell mediated cytotoxicity (NES 1.71), and this is noteworthy because memory CD8 T cells can gain NK-like functional responses (46). Hurdle analysis of activated CD8 T cells identified 27 DEGs; following Immune Education, expression in 10 was upregulated while expression in 17 was downregulated (**Figure 4D**). Among the genes whose expression were affected by Immune Education were *Klrc1* (*Nkg2a/b*; upregulated 3.22 fold, P<0.0001) and *Klrk1* (*Nkg2d*; upregulated 3.33 fold, P=0.0012), whose encoded proteins are members of the NKG2 (CD159) family of C-type lectin-like receptors. This is notable because these proteins complex with CD94 to form a heterodimer crucial to CD8 T cell survival and function (47–49). In activated CD8 T cells, Immune Education also affected expression of *Nfkbia* (downregulated 2.07 fold, P=0.0013), a trend also observed in the naïve CD8 T cell subset, indicating increased CD8 T cell proliferation after Immune Education (35).

For memory CD4 T cells (cluster 5), GSEA showed 1 upregulated KEGG pathway after Immune Education, the alanine, aspartate, and glutamate metabolism pathway (NES 1.84). This may reflect the metabolic demands induced by differentiation of memory CD4 T cell into activated effector cells (50). Hurdle analysis of memory CD4 T cells identified 28 DEGs; following Immune Education, expression in 19 was upregulated while expression in 9 was downregulated (**Figure 4E**). Among the genes whose expression were affected by Immune Education was *Gadd45b* (downregulated 2.20 fold, P<0.0001), a member of the growth arrest and DNA damage (GADD)-inducible gene family. GADD45B enhances T cell survival by blocking Fas-induced apoptosis to inhibit activation induced cell death (AICD); however, *Gadd45b* mRNA expression in response to stimuli has previously been shown to rapidly peak and then decrease (51, 52). This is notable given upregulation of *Il18r1* (upregulated 2.34 fold, P=0.0076) and *Ifng* (upregulated 2.02 fold, P=0.0159) following Immune Education because synergistic IL-12 and IL-18 induction of *Gadd45b*, independent of TCR signaling, has been shown to promote IFN-γ production by Th1 T cells (53, 54).

The cytotoxic CD8 T cell subset (cluster 6) in Immune-Educated mice when compared to Controls had 21 upregulated KEGG pathways including antigen processing and presentation (NES 1.74) which is essential to the cytotoxic capacity of CD8 T cells (55). Hurdle analysis of cytotoxic CD8 T cells revealed 29 DEGs; following Immune Education, expression in 13 were upregulated while expression in 16 were downregulated (**Figure 4F**). Among the genes whose expression were affected by Immune Education was *Ilf2* (*Nf45*; upregulated 4.42 fold, P=0.0048), whose encoded protein is part of the nuclear factor of activated T cells (NFAT) complex which acts as a transcription factor to induce T cell expression of various genes, including IL2 (56). Among the genes whose expression were downregulated following by Immune Education was *Nkg7* (downregulated 4.78 fold, P=0.0012), which is an important gene for the efficiency of CD8 T cell mediated cytotoxicity. Furthermore, downregulation of *Nkg7* in the cytotoxic CD8 T cell subset from Immune-Educated mice may represent a memory phenotype rather than an effector phenotype (57).

Additionally, the DEGs from the CD4^+^ and CD8^+^ subsets were queried for overlapping genes. As shown in **Figure 4G**, there was one gene in common between DEGs from quiescent naïve CD4 and memory CD4 T cells and two genes in common between DEGs from primed naïve CD4 and memory CD4 T cells. Notably, *Nfkbia* was differentially expressed in both primed naïve CD4 and memory CD4 T cells which underscores its role in CD4 T cell proliferation and survival (35, 36). As shown in **Figure 4H**, there was one gene in common between all CD8 subsets, two genes in common between naïve and activated CD8 T cells, and one gene in common between naïve CD8 and cytotoxic CD8 T cells. The gene in common between all CD8 subsets was *Nfkbia*, which is also essential to CD8 T cell proliferation (35). Additionally, *Cd48* was upregulated in naïve CD8 and activated CD8 T cells suggesting enhanced priming of and cytotoxic capacity for CD8 T cells in mice after Immune Education (39).

### 3.4 Analysis of the genomic changes in CLP mice versus control mice revealed a disorganized T cell immune response to CLP

A list of all significant KEGG pathways in CLP mice relative to Control mice across the T cell subsets can be found in **Supplementary Table 4**. A list of all significant DEGs in CLP mice relative to Control mice across the T cell subsets can be found in **Supplementary Table 5**.

Quiescent naïve CD4 T cells in CLP mice had 85 significant KEGG pathways with 1 upregulated and 84 downregulated when compared to those in Controls. Among the gene sets affected by CLP was the IL17 signaling pathway (NES -1.75), which is significant because IL17 is a key cytokine for the host innate immune response to mucosal fungal and bacterial infections (58). Following CLP, the MAPK signaling KEGG pathway (NES -1.76) was also downregulated, and this is notable because MAPK signaling has been implicated in TCR mediated naïve CD4 T cell clonal expansion (59). Hurdle analysis of quiescent naïve CD4 T cells identified 131 DEGs; following CLP, expression in 47 was upregulated while expression in 84 was downregulated. Among the genes whose expression were affected by CLP was *Dtx1* (upregulated 4.48 fold, P<0.0001), which is notable because it favors Th17 differentiation following IL6 and TGFβ treatment *in vitro* (60). Another gene whose expression was affected by CLP was *Socs3* (upregulated 4.48 fold, P<0.0001), whose encoded protein has been shown to promote Th17 cell differentiation, reduce IL2 production, and possibly prevent further CD4 T cell proliferation (61).

GSEA of primed naïve CD4 T cells revealed 57 significant KEGG pathways, all downregulated, in CLP mice relative to Controls. Hurdle analysis of primed naïve CD4 T cells identified 97 DEGs; following CLP, expression in 30 was upregulated while expression in 67 was downregulated. Among the genes whose expression were affected by CLP were *Dtx1* (upregulated 3.31 fold, P=0.0008) and *Socs3* (upregulated 6.45 fold, P<0.0001), similar to changes seen in the quiescent naïve CD4 T cell subset. Additionally, following CLP there was upregulation of *Il7r* (upregulated 4.55 fold, P=0.0001) without corresponding upregulation in downstream members of the *Bcl2* family indicating a lack of downstream IL7 signaling in the primed naïve CD4 T cells. Rather, there was downregulation of the *Bcl2* family member *Bcl11b* (downregulated 3.04 fold, P=0.0077) following CLP. Bcl11b is a T-lineage commitment factor essential to thymocyte development and the positive selection of both CD4 and CD8 single positive thymocytes (62). Moreover, in CD4 T cells Bcl11b is crucial for the induction of *Foxp3* expression in response to TGF-β signaling during peripheral T_reg_ proliferation (62, 63). Bcl11b also promotes CD4 T cell differentiation into Th17 cells by repressing *Gata3* expression to restrict Th2 lineage proliferation (62, 64). Overall, it was difficult to assess the DEGs between naïve CD4 T cells from CLP and Control mice, possibly due to a disorganized T cell immune response characterized by predominantly naïve T cell populations.

In the naïve CD8 T cell subset, GSEA revealed 169 significant KEGG pathways with 9 upregulated and 160 downregulated in CLP mice compared to Controls. Among the affected gene sets was the PD-L1 expression and PD-1 checkpoint pathway in cancer KEGG pathway (NES -2.06), which is significant because it has been suggested that PD-L1 is a negative regulator of effector CD8 T cell proliferation and response to pathogens *in vivo* (65). However, concomitant downregulation of the TCR signaling pathway (NES -2.04) following CLP suggests a disorganized T cell immune response in the CLP mice. Hurdle analysis of naïve CD8 T cells identified 207 DEGs; following CLP, expression in 29 was upregulated while expression in 178 was downregulated. Among the genes whose expression were affected by CLP was *Ly6a* (*Sca1*; upregulated 10.17 fold, P<0.0001), whose encoded protein is a well-established marker of murine hematopoietic stem cells known to be upregulated on naïve CD8 T cells (66, 67). Ly6a has been shown to be highly expressed during viral-mediated memory CD8 T cell development (68); however, in a murine knockout model, it was demonstrated that *Ly6a* is not required for the development of competent memory CD8 T cells (66). Other previously discussed genes that were upregulated in the naïve CD8 T cells following CLP include *Dtx1* (upregulated 3.02 fold, P<0.0001), *Runx3* (upregulated 2.66 fold, P<0.0001), *Cd7* (upregulated 2.57 fold, P<0.0001), and *Socs3* (upregulated 2.15 fold, P=0.0002). Among the genes downregulated following CLP in the naïve CD8 subset was *Lgals1* (downregulated 2.92 fold, P<0.0001), whose encoded protein galectin-1 is secreted by CD8 T cells to antagonize persistent TCR agonism and tune the CD8 immune response (69). Downregulation of *Lgals1* suggests susceptibility to a dysregulated CD8 T cell response following CLP. Additionally, in naïve CD8 T cells the expression of *Cd28* (downregulated 3.52 fold, P<0.0001) was affected post-CLP, and this is important because *Cd28* is essential to CD8 survival following an acute immune response (70). Other previously discussed genes that were downregulated in the naïve CD8 T cells following CLP include *Nfkbia* (downregulated 3.89 fold, P<0.0001) and *Bcl11b* (downregulated 2.14 fold, P=0.0084).

For activated CD8 T cells there were 97 significant KEGG pathways with 2 upregulated and 95 downregulated in CLP mice when compared to Control mice. Hurdle analysis of activated CD8 T cells identified 125 significant DEGs; following CLP, expression in 46 was upregulated while expression in 79 was downregulated. Among the genes whose expression were affected by CLP was *Il7r* (upregulated 2.55 fold, P <0.001); however, there was no upregulation of downstream *Bcl2* family members suggesting a lack of IL7 signaling in this T cell subset. In fact, there was downregulation of the Bcl-2 family member *Mcl1* (downregulated 2.46 fold, P <0.001) following CLP. Mcl-1 has been shown to antagonize Bim, a pro-apoptotic member of the Bcl-2 protein family, to prevent effector CD8 T cell contraction following acute antigen-driven proliferation and promote activated CD8 T cell proliferation into memory CD8 T cells (71, 72). Additionally, there was downregulation of *Xcl1* (downregulated 3.96 fold, P=0.0007) following CLP. Xcl-1 (lymphotactin) is released by activated CD8 T cells and binds to Xcr-1 to recruit cross-presenting dendritic cells that enhance cytotoxic CD8 T cell function and promote antigen-specific memory CD8 T cell proliferation (73, 74). These results suggest impaired effector and memory CD8 T cell expansion in CLP mice compared to Control mice.

The memory CD4 T cells from CLP when compared to Control had 76 significant KEGG pathways with 1 upregulated and 75 downregulated. Hurdle analysis of memory CD4 T cells identified 123 DEGs; following CLP, expression in 45 was upregulated while expression in 78 was downregulated. Notably, following CLP there was upregulation in the expression of *Il7r* (upregulated 5.57 fold, P<0.0001) and *Bcl2* (upregulated 2.79 fold, P=0.0019). This points towards development of a memory CD4 T cell pool following exposure to CLP.

In the cytotoxic CD8 T cell subset there were 24 significant KEGG pathways, all upregulated, in the CLP mice when compared to Controls. Hurdle analysis of cytotoxic CD8 T cells identified 56 DEGs; following CLP expression in 25 was upregulated while expression in 31 was downregulated. Among the upregulated genes in cytotoxic CD8 T cells following CLP was *Ly6a* (upregulated 11.35 fold, P<0.0001). Among the downregulated genes in cytotoxic CD8 T cells following CLP were *Lgals1* (downregulated 6.33 fold, P=0.0058), *Bcl10* (downregulated 3.87 fold, P=0.0019), and *Nfkbia* (downregulated 2.50 fold, P=0.0093).

### 3.5 Transcriptomic changes in immune-educated CLP mice versus immune-educated mice demonstrated distinct CD4 and CD8 T cell responses to CLP

A list of all significant KEGG pathways in Immune-Educated CLP mice relative to Immune-Educated mice across the T cell subsets can be found in **Supplementary Table 6**. A list of all significant DEGs in Immune-Educated CLP mice relative to Immune-Educated mice across the T cell subsets can be found in **Supplementary Table 7**.

**Figure 5:**
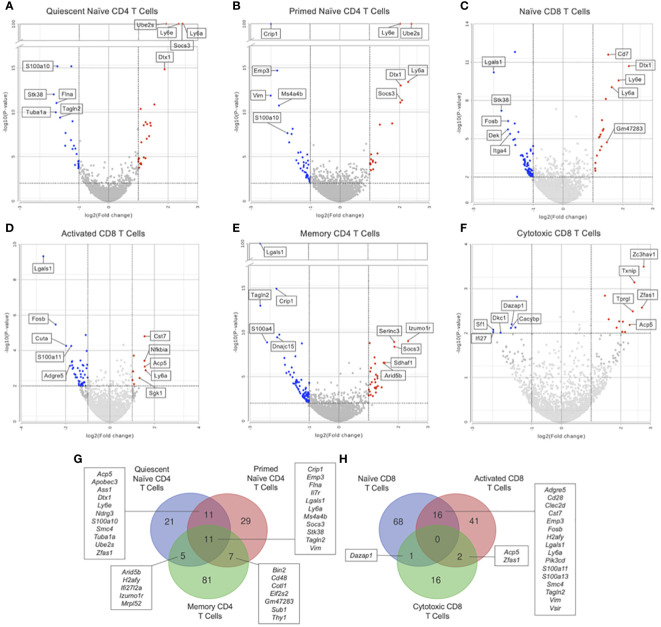
Hurdle analysis was performed within each T cell subset to see if there were differentially expressed genes in Immune-Educated CLP mice relative to Immune-Educated mice. Volcano plots were used to show the adjusted P values and log2 fold change values from these analyses. Red dots indicate upregulated genes, blue dots indicate downregulated genes, and gray dots represent insignificant or inconclusively different genes in Immune-Educated CLP mice relative to Immune-Educated mice. The top 5 upregulated and top 5 downregulated genes are labeled on the volcano plots. Results for **(A)** quiescent naïve CD4, **(B)** primed naïve CD4, **(C)** naïve CD8, **(D)** activated CD8, **(E)** memory CD4, and **(F)** cytotoxic CD8 T cells are shown. The full list of DEGs can be found in **Supplementary Table 7**. Overlapping DEGs in the **(G)** CD4^+^ subsets and **(H)** CD8^+^ subsets were identified and depicted on Venn diagrams.

Within the quiescent naïve CD4 T cell subset, GSEA revealed 79 upregulated pathways in Immune-Educated CLP mice when compared to Immune-Educated mice. Affected KEGG pathways included antigen processing and presentation (NES 1.60), IL-17 signaling pathway (NES 1.57), and TCR signaling (NES 1.37) suggestive of a coordinated T cell response to CLP in quiescent naïve CD4 T cells following Immune Education. Hurdle analysis of quiescent naïve CD4 T cells identified 48 DEGs; following Immune Education and CLP versus Immune Education alone, expression in 24 was upregulated while expression in 24 was downregulated (**Figure 5A**). Among the upregulated genes was *Socs3* (upregulated 5.56 fold, P<0.0001), which suggests a Th17-skewed naïve CD4 T cell response to CLP (75). There was also upregulation of *Il7r* (upregulated 2.13 fold, P<0.0001), but no upregulation in downstream members of the *Bcl2* family to indicate IL7-mediated signaling in the quiescent naïve CD4 T cells. Another upregulated gene in quiescent naïve CD4 T cells from Immune-Educated CLP mice was *Dtx1* (upregulated 3.70 fold, P<0.0001), which is a downstream transcription factor induced by NFAT signaling that promotes T cell anergy (76). Together these results suggest an immune state favoring naïve CD4 T cell contraction following antigen-mediated TCR signaling.

For primed naïve CD4 T cells, GSEA identified 64 KEGG pathways of significance with 1 upregulated and 63 downregulated from the Immune-Educated CLP mice relative to Immune-Educated mice. Hurdle analysis of primed naïve CD4 T cells identified 58 DEGs; following Immune Education and CLP versus Immune Education alone, expression in 19 was upregulated while expression in 39 was downregulated (**Figure 5B**). Like in the quiescent naïve CD4 T cell subset, there was upregulation of *Socs3* (upregulated 4.23 fold, P<0.0001) and *Dtx1* (upregulated 4.11 fold, P<0.0001) in primed naïve CD4 T cells from Immune-Educated CLP mice relative to those from Immune-Educated mice. Also, there was once again upregulation of *Il7r* (upregulated 4.08 fold, P<0.0001) in primed naïve CD4 T cells from Immune-Educated CLP mice without change in downstream *Bcl2* family gene expression. These results are again suggestive of conditions favoring naïve CD4 T cell contraction following T cell signaling.

GSEA of naïve CD8 T cells from Immune-Educated CLP mice versus Immune-Educated mice revealed 96 significant KEGG pathways with 2 upregulated and 94 downregulated. Hurdle analysis of naïve CD8 T cells identified 85 DEGs; following Immune Education and CLP versus Immune Education alone, expression in 18 was upregulated while expression in 67 was downregulated (**Figure 5C**). Previously discussed genes that were upregulated in the naïve CD8 T cells from Immune-Educated CLP mice include *Dtx1* (upregulated 4.62 fold, P<0.0001), *Ly6a* (upregulated 3.10 fold, P<0.0001), *Cd7* (upregulated 2.86 fold, P<0.0001), and *Socs3* (upregulated 2.30 fold, P<0.0001). There was again upregulation of *Il7r* (upregulated 2.12 fold, P=0.0032) without corresponding upregulation in *Bcl2* family members. Among the downregulated genes in naïve CD8 T cells from Immune-Educated CLP was *Lgals1* (downregulated 5.00 fold, P<0.0001), whose encoded protein galectin-1 tunes the CD8 immune response as previously discussed (69). Moreover *Cd5* (downregulated 2.54 fold, P=0.0002) and *Cd28* (downregulated 2.39 fold, P<0.0025), genes encoding two previously discussed cell surface molecules, were downregulated in naïve CD8 from Immune-Educated CLP mice when compared to Immune-Educated mice. Together, these findings point towards a dysregulated naïve CD8 response with impaired CD8 T cell expansion in response to CLP in mice that underwent Immune Education.

In the activated CD8 T cell subset, 57 significant KEGG pathways were identified on GSEA of Immune-Educated CLP versus Immune-Educated with 7 upregulated and 50 downregulated. Hurdle analysis of activated CD8 T cells identified 59 DEGs; following Immune Education and CLP versus Immune Education alone, expression in 9 was upregulated while expression in 50 was downregulated (**Figure 5D**). Previously discussed genes that were upregulated in activated CD8 T cells from Immune-Educated CLP mice included *Nfkbia* (upregulated 2.93 fold, P=0.0004) and *Ly6a* (upregulated 3.04 fold, P=0.0013). Among the downregulated genes in activated CD8 T cells from Immune-Educated CLP mice was *Il18r1* (downregulated 3.21 fold, P=0.0011), which is upregulated in effector CD8 T cells during acute viral infection and downregulated in exhausted CD8 T cells during chronic infection (77). Other previously discussed genes that were downregulated in the activated CD8 T cells from Immune-Educated CLP mice include *Lgals1* (downregulated 8.01 fold, P<0.0001) and *Cd28* (downregulated 3.26 fold, P=0.0007). These results are suggestive of an acute CD8 response to CLP which may be dysregulated in Immune-Educated mice.

In the memory CD4 T cell subset there were 91 downregulated KEGG pathways in Immune-Educated CLP when compared Immune-Educated. Hurdle analysis of memory CD4 T cells identified 104 DEGs; following Immune Education and CLP versus Immune Education alone, expression in 28 was upregulated while expression in 76 was downregulated (**Figure 5E**). One of the upregulated genes was *Izumo1r* (upregulated 5.04 fold, P<0.0001), which is expressed in *Foxp3* ^+^ T_reg_ cells and was recently shown to facilitate T_reg_ tight contacts with γδT cells to mediate psoriasis-like inflammation in the dermis (78). There was also upregulation of *Socs3* (upregulated 3.66 fold, P<0.0001), which implies a Th17-skewed environment (61). Additionally, there was upregulation of *Il7r* (upregulated 2.33 fold, P<0.0001). Previously discussed genes that were downregulated in the memory CD4 T cell subset from Immune-Educated CLP mice include *Lgals1* (downregulated 6.27 fold, P<0.0001) and *Il18r1* (downregulated 3.01 fold, P<0.0001).

Cytotoxic CD8 T cells from Immune-Educated CLP mice had 3 upregulated KEGG pathways relative to Immune-Educated mice. Hurdle analysis of cytotoxic CD8 T cells identified 19 DEGs; following Immune Education and CLP versus Immune Education alone, expression in 12 was upregulated while expression in 7 was downregulated (**Figure 5F**). Among the upregulated genes was *Pdcd4* (upregulated 2.76 fold, P=0.0014), whose encoded protein has been implicated in T cell exhaustion and apoptosis (79).

Additionally, the DEGs from the CD4^+^ and CD8^+^ subsets were searched for overlapping genes. As shown in **Figure 5G**, there were eleven genes in common between all CD4, eleven genes in common between quiescent naïve CD4 and primed naïve CD4, five genes in common between quiescent naïve CD4 and memory CD4, and seven genes in common between primed naïve CD4 and memory CD4 T cells. Notably *Il7r*, which plays a central role in T cell proliferation and survival was differentially expressed in all CD4 subsets (13). As shown in **Figure 5H**, there were sixteen genes in common between naïve CD8 and activated CD8, one gene in common between naïve CD8 and memory CD8, and two genes in common between naïve and activated CD8 T cells. Among the genes differentially expressed in both naïve CD8 and activated CD8 T cells was *CD28* (downregulated in both subsets), which plays a role in effector CD8 T cell survival following activation (70), and *Lgals1* (downregulated in both subsets) which encodes galectin-1, a protein released by CD8 T cells to antagonize persistent TCR activation (69). No gene was differentially expressed in all CD8 subsets.

### 3.6 Immune education prior to CLP enhanced TCR signaling with transcriptomic signatures suggesting improved T cell survival and function

A list of all significant KEGG pathways in Immune-Educated CLP mice relative to CLP mice across the T cell subsets can be found in **Supplementary Table 8**. A list of all significant DEGs in Immune-Educated CLP mice relative to CLP mice across the T cell subsets can be found in **Supplementary Table 9**.

**Figure 6:**
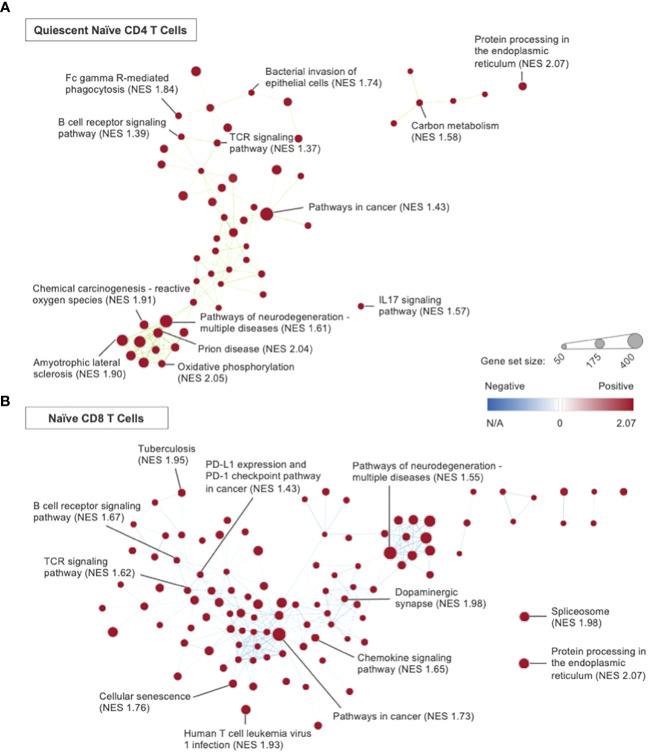
Network analysis, using Cytoscape, was performed using the GSEA results from the Immune- Educated CLP versus CLP experimental group comparison. Representative plots were created for the **(A)** quiescent naïve CD4 T cell and **(B)** naïve CD8 T cell subsets. Each node represents a KEGG gene set, with node sizes representative of the gene set size and node color representative of the degree of normalized enrichment. Nodes are connected by edges, which are determined by the degree of overlapping genes between nodes. Gene sets with the 5 highest NES within each T cell subset and others of interest were labeled.

In the quiescent naïve CD4 T cell subset, GSEA identified 79 KEGG pathways of significance with all upregulated in Immune-Educated CLP mice when compared to CLP mice. Network analysis using GSEA results (**Figure 6A**) demonstrated an edge—a connection between gene set nodes based on the degree of overlapping genes—connecting the TCR (NES 1.37) and B cell receptor (NES 1.39) signaling pathways. This indicates overlap between these two signaling pathways. Among the genes within the edge were *Nfatc1* and *Nfatc2*, which encode transcription factors necessary for both T cell and B cell survival (35, 80, 81). Hurdle analysis of quiescent naïve CD4 T cells identified 31 DEGs; following Immune Education and CLP versus CLP alone, expression in 24 was upregulated while expression in 7 was downregulated. Among the affected genes in the Immune-Educated CLP mice was *Bsg* (upregulated 2.29 fold, P <0.001), whose encoded protein basigin/CD147 has been shown to play a role in T cell activation and T_reg_ differentiation, and because of this it has been studied as a potential therapeutic target for inflammatory diseases (82, 83). There was also upregulation of *Cdc42* (upregulated 2.25 fold, P <0.001), whose encoded protein (a Rho family GTPase) has been shown to play an essential role in actin polymerization for TCR clustering, Th1 differentiation, and memory cell survival in naïve CD4 T cells (84). Another gene whose expression was upregulated following Immune-Educated CLP was *Rac1* (upregulated 2.22 fold, P <0.001), which encodes another Rho family GTPase that plays a critical role in CD4 single-positive T cell differentiation by preventing TCR-induced apoptosis via *Bcl2* upregulation (85). Among the downregulated genes was *Zbp1* (downregulated 3.44 fold, P <0.001), whose encoded protein is an important immune sensor critical to initiation of the innate immune response to pathogens. However, excessive *Zbp1* mediated PANoptosis has been shown to have detrimental host effects, thus *Zbp1* signaling may play a critical role in immune homeostasis (86). Together these results suggest enhanced naïve CD4 TCR signaling in response to CLP following Immune Education.

In the primed naïve CD4 T cell subset there were 60 significant KEGG pathways that were all upregulated with Immune-Educated CLP relative to CLP. Hurdle analysis of primed naïve CD4 T cells identified 24 DEGs; following Immune Education and CLP versus CLP alone, expression in 22 was upregulated while expression in 2 was downregulated. Two of the upregulated genes were *Lck* (upregulated 2.39 fold, P <0.001) and *Cd2* (upregulated 2.26 fold, P<0.001). Lck is a member of the Src family of protein tyrosine kinases, and it is involved in TCR phosphorylation which regulates initiation of TCR signaling and T cell development (87). CD2 has been shown to interact with Lck as a co-stimulatory molecule in T cell activation (88). Additionally, there was upregulation of *Cdc42* (upregulated 2.14 fold, P=0.0012), which may again indicate enhanced naïve CD4 TCR signaling in Immune-Educated CLP mice when compared to CLP mice.

For naïve CD8 T cells from Immune-Educated CLP mice, GSEA revealed 128 upregulated pathways relative to those from CLP mice. Network analysis using GSEA results (**Figure 6B**) demonstrated an edge connecting the TCR signaling pathway (NES 1.62) and PD-L1 expression and PD-1 checkpoint pathway in cancer (NES 1.43) – these processes are likely connected given the acute upregulation of PD-1 in response to TCR activation (89, 90). Hurdle analysis of naïve CD8 T cells identified 77 DEGs; following Immune Education and CLP versus CLP alone, expression in 68 was upregulated while expression in 9 was downregulated. One of the upregulated genes was *Nkg7* (upregulated 2.66 fold, P<0.0001), whose encoded protein has been shown to improve the efficiency of CD8 T cell synapse formation to enhance cytotoxic effects and limit inflammation (57). Other previously discussed genes that were upregulated with Immune-Educated CLP relative to CLP include *Cd7* (upregulated 2.69 fold, P=0.0019), *CD28* (upregulated 2.51 fold, P=0.0034), and *Mcl1* (upregulated 2.34 fold, P=0.0088). These findings indicate enhanced naïve CD8 T cell survival for mice exposed to CLP after Immune Education. Among the downregulated genes was *Pdcd6* (*Alg2*; downregulated 2.44 fold, P=0.0043), whose encoded protein plays a role in TCR mediated programmed cell death by affecting the stability of Mcl1 following T cell activation to promote apoptosis (91). These results suggest enhanced survival of naïve CD8 T cells post-CLP following Immune Education versus without.

GSEA of activated CD8 T cells revealed 10 upregulated KEGG pathways in Immune-Educated CLP mice versus CLP mice. Hurdle analysis of activated CD8 T cells identified 37 DEGs; following Immune Education and CLP versus CLP alone, expression in 17 was upregulated while expression in 20 was downregulated. Among the upregulated genes was *Klrc1* (*Nkg2a*; upregulated 2.89 fold, P=0.0041). *Klrc1* has been shown to be inversely correlated with CD8 T_reg_ function in anti-CD3 mAb stimulated human CD8 T cells (92). *Nfkbia* expression was again affected (upregulated 3.15 fold, P=0.0027) indicating enhanced CD8 T cell immune function (93). Among the downregulated genes in activated CD8 T cells following Immune-Educated CLP versus CLP was *Slamf6* (downregulated 2.35 fold, P=0.0006), whose encoded protein is a CD2 family member that has been implicated in CD8 exhaustion. It has been shown that anti-SLAMF6 could correct CD8 dysfunction in leukemias and lymphomas (94).

Within the memory CD4 T cell subset, GSEA revealed 48 upregulated pathways in Immune-Educated CLP compared to CLP. Hurdle analysis of memory CD4 T cells identified 46 DEGs; following Immune Education and CLP versus CLP alone, expression in 28 was upregulated while expression in 18 was downregulated. One of the upregulated genes was *Txk* (upregulated 2.35 fold, P=0.0040), which encodes a member of the Tec family of non-receptor tyrosine kinases. It has been suggested that *Txk* may have a role in potentiating Th2 function in memory T cells (95). One of the downregulated genes was *Il7r* (downregulated 2.78 fold, P=0.0037).

In cytotoxic CD8 T cells from Immune-Educated CLP mice, GSEA revealed 5 significant pathways with 1 upregulated and 4 downregulated relative to those from CLP mice. Hurdle analysis of cytotoxic CD8 T cells identified 12 DEGs; following Immune Education and CLP versus CLP alone, expression in 10 was upregulated while expression in 2 was downregulated.

### 3.7 Immune Education upregulated T cell IL7R expression in mice exposed to CLP

**Figure 7:**
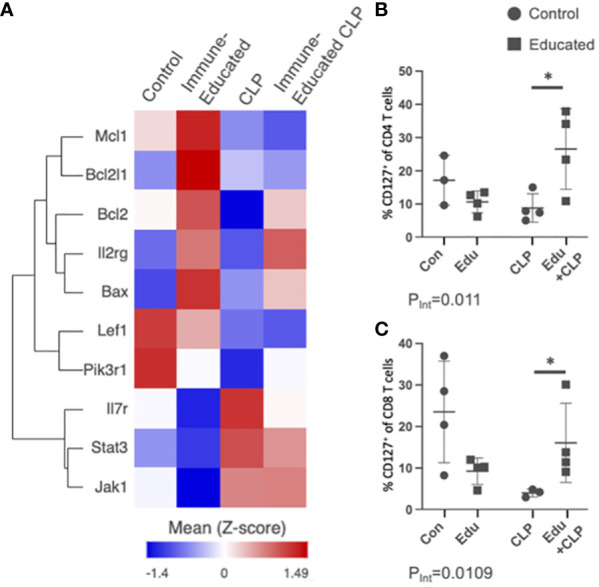
**(A)** Using the scRNA-seq data, a heat map of genes involved in the IL7R pathway across all T cells by experimental group was created for visualization. Dot plots were created to display the percent of IL7R/CR127^+^ T cells on flow cytometric analysis for **(B)** CD4 T cells and **(C)** CD8 T cells across the experimental groups (Con=Control, Edu=Immune-Educated, CLP=CLP, and Edu+CLP=Immune-Educated CLP) to corroborate scRNA-seq findings of increased IL7R/CD127 expression following CLP and Immune-Educated CLP. It was shown that Immune Education increased the proportion of CD127^+^ CD4 and CD8 T cells when exposed to CLP.

Several of the subpopulations in the Immune-Educated CLP mice in our scRNA-seq analysis demonstrated upregulation of the IL7R signaling pathway. **Figure 7A** shows a heat map of genes involved in the IL7R (CD127) pathway across all T cells by experimental group. There was upregulation of *Il7r* for CLP and Immune-Educated CLP mice, but *Il2rg*, *Bcl2l1*, and *Bax* were only upregulated in the Immune-Educated mice. Additionally, *Bcl2* was upregulated with Immune-Educated and Immune-Educated CLP, but not CLP. *Mcl1* was also upregulated in Immune-Educated CLP. The Bcl-2 family of proteins are located on the outer mitochondrial membrane and control mitophagy by regulating voltage-dependent anion channels to influence mitochondrial Ca^2+^ signaling (96). Bax and Bak are proapoptotic members of the Bcl-2 family essential to mitochondrial-dependent apoptotic pathways in multiple cell types, including T cells (97). We performed flow cytometry to validate these finding in our mouse model and found that Immune Education had a statistically significant effect on IL7R (CD127) expression in mice subjected to CLP for CD4 (P_Int_=0.0110; **Figure 7B**) and CD8 T cells (P_Int_=0.0109; **Figure 7C**).

These findings are significant given the role of IL7 in T cell survival. A prior study has demonstrated that recombinant human IL7 (rhIL7) treatment 24-48 hours after CLP exposure blocks CD4 and CD8 T cell apoptosis in mice following CLP exposure by increasing Bcl-2 (13). This has led to trials investigating the effect of IL7 in human sepsis (13, 14). The IL7R pathway is one example of how findings from this study can be applied to other investigations.

## 4 Conclusion

Following antigenic stimulation via the TCR there is expansion and differentiation of activated T cells. After clearance of a pathogen, a balanced immune response will eliminate excess effector T cells while generating a memory T cell pool (98). One of the major limitations in translating work from murine sepsis models to humans has been the lack of memory T cell compartments in mice that are bred in sterile facilities (6). There are several highly salient points that arise from the current work. Most importantly, we identify that T cell responses following CLP are not homogenous. Our work identifies several subsets of naïve and memory CD4 and CD8 T cells that likely have variable responses and functionally contribute differing factors to the overall response to CLP. The same is likely true of the human T cell response to sepsis. To date though, very little has been done examining differential T cell subset responses in human sepsis. It is theorized that some T cells in sepsis are directed against the sepsis-inciting pathogen while others are inappropriately activated, and still others are undergoing apoptosis, T_reg_ cell differentiation, or other types of elimination. The variation in T cell subset responses to sepsis and severe inflammation deserves further examination in future studies.

The role of T cell memory in modulation of the T cell immune response also warrants further discussion. Immune Education in our model promoted widespread changes in many pathways following CLP, indicating that T cell memory is a major factor in modulation of the immune response to major infection and likely to human sepsis. Several seminal papers have argued that the immune response in mice, using transcriptomic methods, fail to emulate the human immune response (99). Similar papers have pointed out, using the same data, that several aspects of the immune response emulate human responses, but the transcriptomic signatures that do not concur have led to major consternation in sepsis research (100). The manuscript by Takao et al. demonstrated that ZAP70 signaling, which is downstream of TCR activation was highly concurrent. This indicates similar TCR signaling between human conditions and murine models; without prior induction of T cell memory, the responses to that TCR signaling in the mouse models may have been drastically different. Our work indicates that T cell memory may have been a driving factor in the malalignment between the murine and human transcriptomic responses discussed in these research studies and this deserves further examination.

There are important limitations to our work and to transcriptomic studies. Single-cell RNA sequencing data lacks the ability to detect functional outcomes – without concurrent protein data, it is difficult to ascertain whether these pathways are leading to cytokine production, changes in cytolytic responses, or alterations in signaling pathways. Further immunologic functional assays are required to substantiate these findings. Much has also been said as to the use of the CLP model. Herein, we used a relatively severe model – this may have biased our results, though it did allow us to demonstrate several important principles that have broad relevance beyond the model itself – and observed responses at a single, early post-CLP time point. The Immune Education method of T cell memory induction is artificial and induces a relatively uniform memory T cell population that may not emulate natural exposures, though in comparison to other T cell “memory induction” methods it does generate broad T cell clonality that is likely closer to the T cell memory in humans. The artificial nature of this induction is balanced by the extreme difficulty of parsing out changes specific to T cell memory and other changes in the innate immune system or the rest of the body caused by natural exposures. Further, work using naturalized mice has many biosafety issues that further limit their use in research studies, making Immune Education a highly accessible and useful model to examine the role of memory T cells in disease models.

Finally, we found several important pathways that may be highly relevant in altering the immune response to CLP. The Th17 response and other T cell effector pathways were upregulated in many T cell subsets in Immune-Educated mice, indicating that T cell memory could alter several other aspects of the CLP response, as we have previously demonstrated (8, 10). We also demonstrate that Immune Education increased expression of surface IL7R and genes downstream of the IL7R signaling pathway upon CLP exposure.

Our use of scRNA-seq uniquely captured the diversity of the full T cell repertoire in immunity. “Bulk” RNA sequencing, which has been the conventional approach to animal and clinical studies assessing T cell response in sepsis, would not allow for the nuanced analysis of T cell subsets performed in this study. However, the scRNA-seq findings presented in this paper are observational in nature and limited because they do not offer mechanistic insights. Future studies will entail utilizing Immune Education prior to CLP to further query mechanisms underlying T cell dysfunction in sepsis.

## Data availability statement

The data presented in the study are deposited in the National Center for Biotechnology Information (NCBI) Sequence Read Archive (SRA) repository, accession number PRJNA1079500. Further inquiries can be directed to the corresponding author.

## Ethics statement

The animal study was approved by Institutional Animal Care and Use Committee (IACUC #2017-039). The study was conducted in accordance with the local legislation and institutional requirements.

## Author contributions

SH: Data curation, Formal analysis, Investigation, Methodology, Validation, Visualization, Writing – original draft, Writing – review & editing. MA: Investigation, Methodology, Writing – review & editing. CD: Conceptualization, Funding acquisition, Methodology, Supervision, Writing – review & editing. MT: Conceptualization, Formal analysis, Funding acquisition, Investigation, Methodology, Validation, Visualization, Writing – original draft, Writing – review & editing.
