# Supplementary figures and images for: Single-cell RNA sequencing reveals Immune Education promotes T cell survival in mice subjected to the cecal ligation and puncture sepsis model
Source: Front Immunol. 2024 Mar 18;15:1366955. doi: 10.3389/fimmu.2024.1366955 (PMC10982361; doi:10.3389/fimmu.2024.1366955)

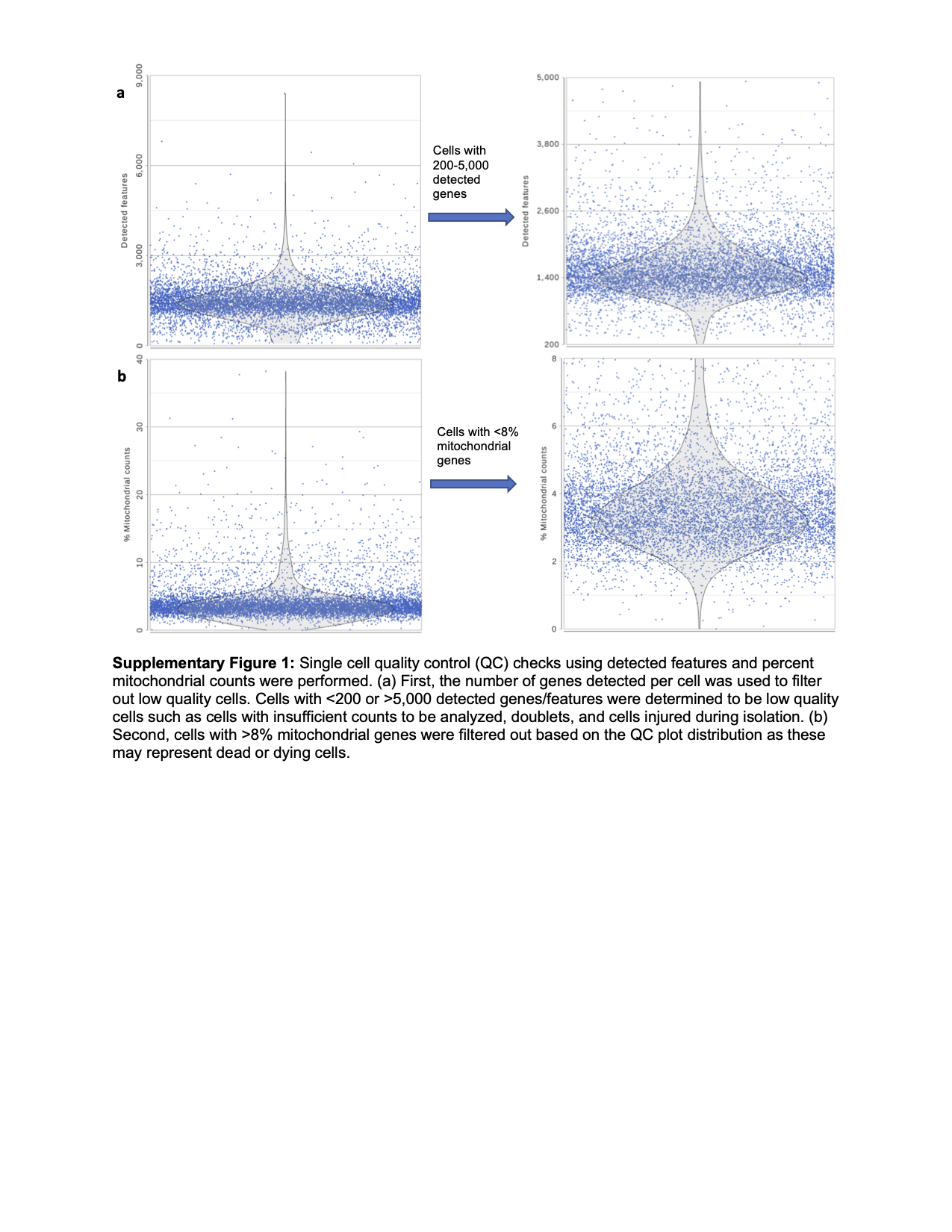

Supplement: Supplementary file 1 [file Image_1.tiff]
