# Supplementary material for: Single-cell RNA sequencing reveals Immune Education promotes T cell survival in mice subjected to the cecal ligation and puncture sepsis model
Source: Front Immunol. 2024 Mar 18;15:1366955. doi: 10.3389/fimmu.2024.1366955 (PMC10982361; doi:10.3389/fimmu.2024.1366955)
Supplement: Supplementary file 2 [file Table_1.pdf]

Quiescent Naïve CD4	Primed Naïve CD4	Naïve CD8	Activated CD8	Memory CD4	Cytotoxic CD8
Igfbp4	Igfbp4	Cd8a	Xcl1	S100a4	Hdac9
Satb1	Ly6c1	Cd8b1	Klrk1	S100a6	Myb
Ly6c1	Npc2	Nkg7	Ccl5	Itgb1	Eomes
Lef1	Pdlim4	Fam241a	Ly6c2	Maf	Slamf7
Foxp1	Stmn1	Plac8	Ctla2a	Tnfrsf4	Kcnc1
Pdlim4	Tesc	Ccr9	Klrd1	S100a11	Pou2af1
Trib2	Ndrg3	Gm43698	Ctsw	Nrp1	Epcam
Cd4	Atp1b1	Lcn4	Nkg7	Ikzf2	Bcat1
Tmem64	Acvrl1	Tmem108	Klrc1	Capg	Tubb2b
Atp1b1	Igsf23	Klrd1	Cd160	Ahnak	Spry1
Smchd1	Aldh2	Itgae	Samd3	Rora	BC035044
Dusp10	Gm12840	Runx3	Hopx	Prr13	Tnfrsf9
Phf20l1	Ramp1	Actn2	Klre1	Cxcr3	Reep1
Bach2	Itm2a	Dnajc15	Klrc2	Izumo1r	Cldn10
Tesc	Lgmn	Dapl1	Klra7	Bcl2a1b	Endou
Clk1	Ggt5	Ctsw	Fcer1g	Eea1	Ptprm
Chd3	Ckb	Bend4	Il2rb	Foxp3	Cd200
Actn1	Trat1	Cd7	Ifitm10	Tbc1d4	Plxdc2
Macf1	Ube2s	Ggt1	Gzmm	Rgs1	Zfpm1
Chd2	Gm37065	Plekho1	Cd7	Casp1	Cd24a
Txnip	Znrf1	Sell	Ms4a4c	Lgals1	Ptprk
St8sia6	Gm15708	Ehd1	Pde2a	Casp4	Itgad
Ndrg3	G430095P16Rik	Epsti1	Ahnak	Smpdl3a	Lad1
4932438A13Rik	Selenop	Tubb5	Il18rap	Icos	Slit3

Supplementary Table 1: Top 25 gene features for each T cell subset identified by graph-based clustering.
